# Modification of Single-Walled Carbon Nanotubes with Halogen-Substituted Metal Phthalocyanines for Chemiresistive Ammonia Detection

**DOI:** 10.3390/ijms27188275

**Published:** 2026-09-17

**Authors:** Darya Klyamer, Pavel Krasnov, Victoria Volchek, Evgeny Maksimovskiy, Anastasiya Fedorenko, Victoria Ivanova, Tamara Basova

**Affiliations:** 1 Nikolaev Institute of Inorganic Chemistry, Siberian Branch of Russian Academy of Sciences, 3, Acad. Lavrentiev Ave., Novosibirsk 630090, Russia; klyamer@niic.nsc.ru (D.K.); volchek@niic.nsc.ru (V.V.); eugene@niic.nsc.ru (E.M.); fedorenko@niic.nsc.ru (A.F.); vivanova@niic.nsc.ru (V.I.); 2 International Research Center of Spectroscopy and Quantum Chemistry, Siberian Federal University, 26 Kirensky St., Krasnoyarsk 660074, Russia; kpo1980@gmail.com

**Keywords:** single-walled carbon nanotubes, metal phthalocyanines, hybrid materials, functionalization, chemiresistive sensors, ammonia detection

## Abstract

The development of highly sensitive and stable gas sensors at room temperature is crucial for environmental and industrial monitoring. This study reports the synthesis and characterization of novel ammonia sensors based on covalently functionalized single-walled carbon nanotubes (SWCNT-NH_2_) with tetra- and octa-chlorinated zinc phthalocyanines (ZnPcCl_4_ and ZnPcCl_8_). These covalent hybrids were prepared by replacing peripheral halogen atoms with amino groups on the surface of the nanotubes, which significantly increased the loading of the macrocycles compared to non-covalently functionalized analogs, reduced nanotube aggregation, and improved electrical conductivity. The sensors exhibited fully reversible responses to NH_3_ at room temperature. Among the tested materials, the covalent hybrid of SWCNT-NH_2_/ZnPcCl_8_ demonstrated superior performance. It achieved a limit of detection of 0.7 ppm and rapid response and recovery times (60 and 65 s, respectively, at 10 ppm). The sensor’s response was over 10 times higher than that of its tetrasubstituted ZnPcCl_4_ counterpart and more than 100 times higher than for non-covalent hybrids. The sensor also showed excellent long-term stability (greater than 1 month) and high selectivity for CO_2_ and volatile organic compounds. Quantum chemical calculations revealed that the increased sensitivity of the octa-chlorinated derivative did not result from stronger analyte binding but rather from the strong electron-withdrawing nature of chlorine substituents, which create an electron-depleted carbon framework. This causes a constant charge transfer from adsorbed NH_3_, inducing a proportionally larger change in majority carrier concentration.

## 1. Introduction

Single-walled carbon nanotubes (SWCNTs) have become a crucial material for the development of next-generation chemical sensing platforms [1,2,3]. Their exceptional charge transport properties, combined with their ultrahigh surface-to-volume ratio, make them highly responsive to the adsorption of various molecules on their surface. However, their effective application as sensing layers is constrained by several factors, including their limited selectivity for target substances, prolonged response and recovery times, and inconsistent performance. One of the main contributors to these issues is the inherent hydrophobicity and strong van der Waals attraction between tubes, which leads to the spontaneous aggregation of tubes into tightly packed bundles. This can hinder the efficient use of SWCNT-based sensors. This bundling severely limits the ability to process the material in a solvent, impedes the formation of uniform thin films, and restricts the diffusion of analytes to the inner surfaces of the tubes, ultimately leading to decreased baseline stability and reduced device-to-device reproducibility. Chemical modification of the nanotube surface offers a robust pathway to mitigate these issues [4]. Through the process of grafting molecular receptors, particularly polyaromatic macrocycles such as pyrenes [5], phenylcoumarin derivatives [6], BODIPYs [7,8], porphyrins [9,10], and phthalocyanines [9,11,12,13], scientists can create hybrid structures that combine the exceptional transduction properties of nanocarbons with the targeted molecular recognition of organic chromophores. In these hybrid materials, the nanotubes serve as a highly conductive communication route, while the attached macrocycles act as specific binding sites. This combination typically results in faster charge transfer, improved analyte discrimination, and better signal reversibility when compared to using each component individually.

Polyaromatic molecules, particularly phthalocyanines, can attach to the surface of nanotubes through both non-covalent and covalent interactions [14,15,16,17]. Non-covalent modifications of nanotubes using π-π stacking, dipole coupling, or hydrogen bonding methods are synthetically straightforward and preserve the sp^2^ lattice structure of the nanotubes. However, physically adsorbed molecules may be susceptible to leaching under conditions of continuous gas flow, repeated cycling, or humidity fluctuations, which can manifest as signal changes. Covalent grafting, on the other hand, creates chemical bonds that firmly attach macrocyclic molecules to the side walls or ends of nanotubes via functional groups. Known methods for covalently functionalizing nanotubes include radical chemistry, cycloadditions, diazonium coupling, nucleophilic substitutions, and reactions with diazonium and dichlorocarbene derivatives [18,19]. For phthalocyanine-based systems, protocols such as formation of amide bonds [20,21,22] and copper-catalyzed azide–alkyne cycloaddition [23,24] have been successfully applied, allowing for the production of structurally robust hybrids.

The electronic properties of phthalocyanines can be modified by introducing halogen atoms (F, Cl) into the outer shell. It has been demonstrated that the energy levels of the highest occupied molecular orbital (HOMO) and the lowest unoccupied molecular orbital (LUMO) decrease as the degree of halogenation increases: MPc > MPcHal_4_ > MpcHal_8_ > MpcHal_16_ [25,26]. The reduction in LUMO enhances electron affinity, making halogen-substituted phthalocyanines more responsive to electron-donating gases like ammonia [27]. However, there are limited studies on the use of halogen-substituted phthalocyanines for the functionalization of carbon nanotubes. Sharma et al. [28,29,30] demonstrated that multi-walled carbon nanotubes (MWCNTs) non-covalently modified with MPcF_16_ (M=Co, Zn) can be used to detect oxidizing gases, such as chlorine. In our recent work [31], we reported chemiresistive sensors based on non-covalent hybrids of pristine SWCNTs and carboxylated SWCNT-COOH with zinc phthalocyanines (ZnPc, ZnPcCl_4_, and ZnPcCl_8_). FTIR, Raman spectroscopy, and inductively coupled plasma atomic emission spectroscopy (ICP-AES) revealed that SWCNT-COOH significantly enhanced phthalocyanine loading via additional hydrogen-bonding interactions. All composites exhibited reversible responses to 1–50 ppm NH3 at room temperature, with ZnPcCl_4_-based hybrids delivering optimal performance. Specifically, SWCNT-COOH/ZnPcCl_4_ achieved a 2–3-fold higher response and a lower limit of detection (0.3 ppm) compared to pristine SWCNT hybrids (0.5 ppm). However, these studies are only sporadic, and the potential of halogenated phthalocyanines for use as covalently bonded receptors in chemiresistive gas sensors has not been fully explored.

To address this knowledge gap, we present a covalent functionalization strategy that couples SWCNTs with two chloro-substituted zinc phthalocyanines (ZnPcCl_x_, x = 4, 8), yielding durable chemiresistive platforms for ammonia monitoring. The hybrids were synthesized via a nucleophilic aromatic substitution reaction between SWCNT-NH_2_ and ZnPcCl_x_, characterized by spectral and electron microscopy techniques, and integrated into planar interdigitated electrode configurations. We systematically evaluate how the identity of the peripheral halogen governs sensor response. The sensor response of the hybrid materials obtained via covalent functionalization of SWCNT-NH_2_ with ZnPcCl_x_ is also compared with similar hybrids obtained via non-covalent functionalization of SWCNT-NH_2_ with ZnPcCl_x_ molecules.

## 2. Results and Discussion

### 2.1. Synthesis and Characterization of Hybrid Materials

Zinc phthalocyanines with four and eight chlorine substituents (ZnPcCl_4_ and ZnPcCl_8_) were used to modify amino-functionalized carbon nanotubes. Commercial amino-functionalized single-walled carbon nanotubes (SWCNT-NH_2_, length 5–30 µm, diameter 1–2 nm, 99%) were used for this reaction. According to the X-ray photoelectron spectroscopy (XPS) analysis, they contain 0.1 at.% nitrogen on their surface.

**Figure 1:**
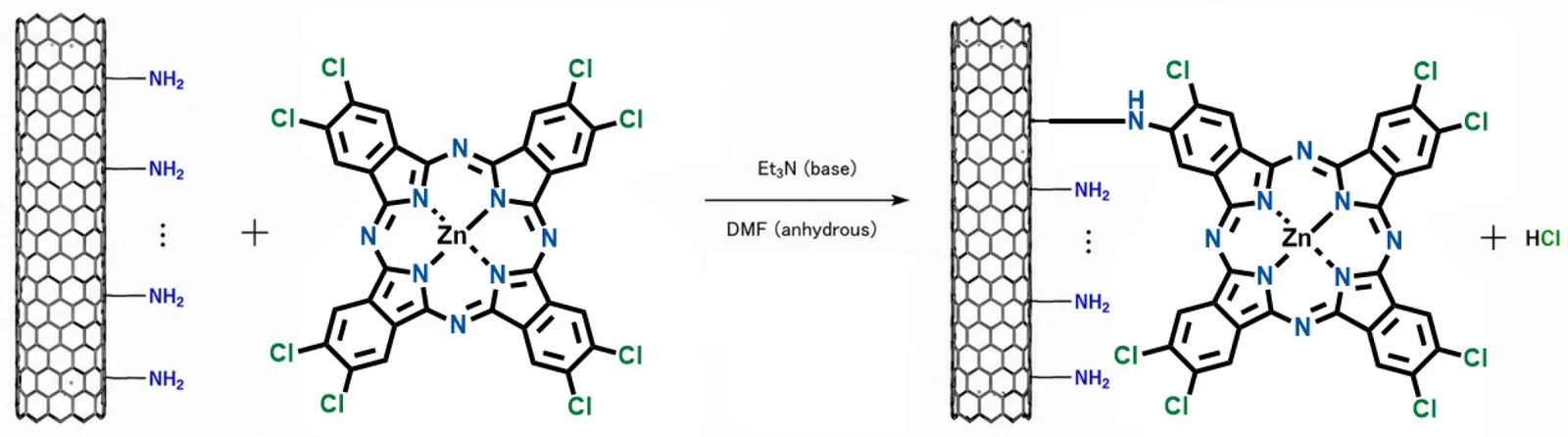
Scheme of the reaction between ZnPcCl_8_ and carbon nanotubes modified with NH_2_.

To create covalent hybrids, a reaction of nucleophilic substitution of peripheral halogen atoms in the phthalocyanine ring with the surface -NH_2_ groups of the nanotubes was employed. This reaction has been previously described for the synthesis of covalent organic frameworks based on MpcHal_x_ [32,33], but it is the first time that it has been applied to the functionalization of carbon nanotubes modified with NH_2_ groups by halogen-substituted phthalocyanines (Figure 1). The reaction conditions were optimized by replacing 1,4-dioxane as the solvent with dimethylformamide (DMF), since syntheses of nanotubes are often carried out using this solvent due to its better dispersibility [20,21,22]. The optimal synthesis time was determined experimentally and was reduced to 24 h. A detailed description of the synthesis procedure can be found in Section 3.1.

**Figure 2:**
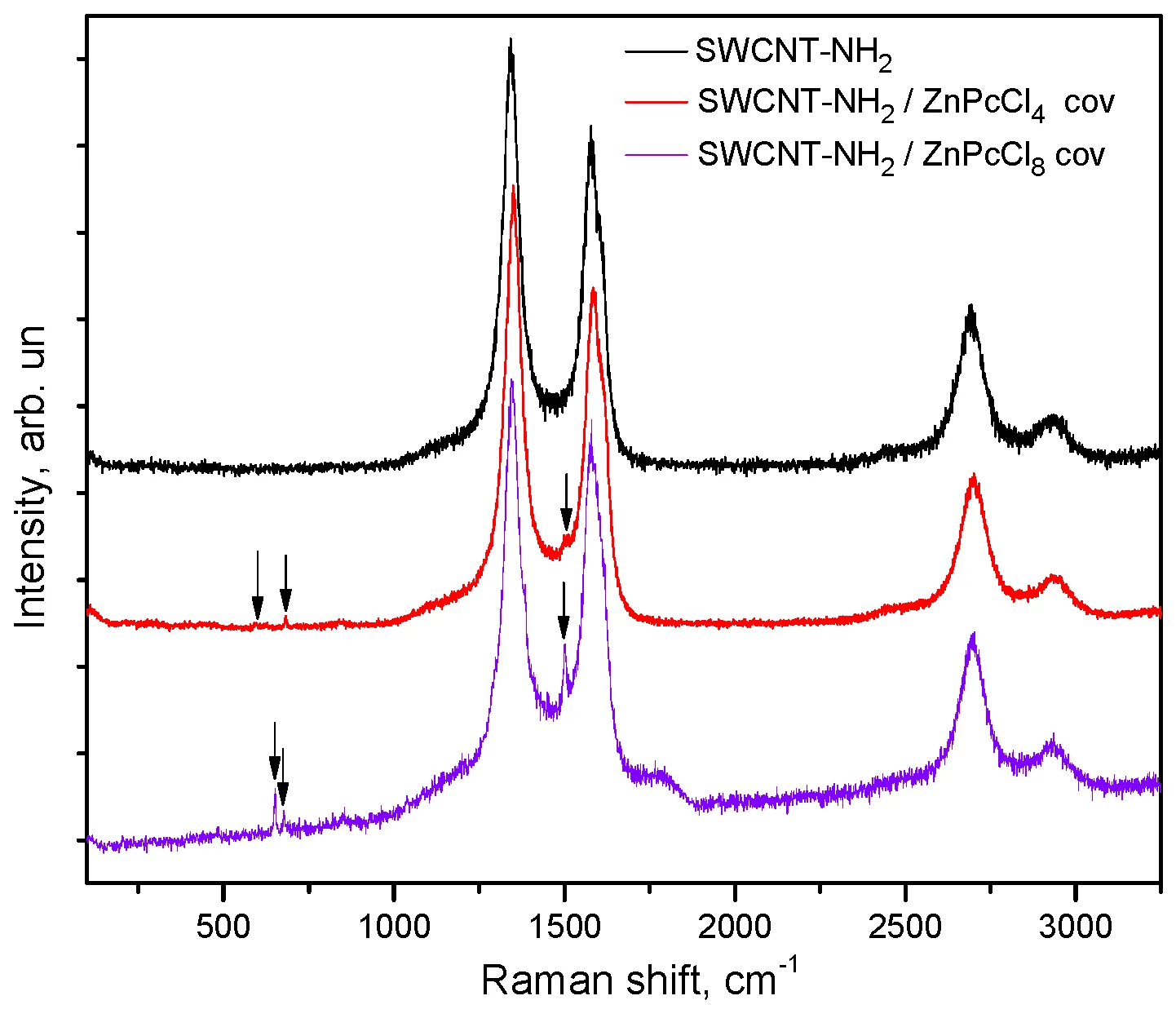
Raman spectra of SWCNT-NH_2_/ZnPcCl_x_ cov. Arrows show the bands corresponding to vibrations of the ZnPcCl_x_ macrocycle.

The resulting hybrids were characterized via Raman spectroscopy, X-ray photoelectron spectroscopy (XPS), scanning electron microscopy (SEM), and ICP-AES. Raman spectra of the SWCNT-NH_2_/MPcCl_x_ cov hybrids are compared with those of pristine SWCNT-NH_2_ in Figure 2. The disordered D and graphite G modes are typical for all carbon nanotubes and their hybrid materials. The G-band is associated with well-ordered sp^2^ carbon and the D-mode with defects or amorphous carbon. The intensity ratio of the D-band and G-band (I_D_/I_G_) is an indicator of the presence of defects in samples. After functionalization of SWCNT-NH_2_ with phthalocyanine molecules, the intensity ratio of the D-band and G-band (I_D_/I_G_), which is an indicator of the presence of defects in nanotubes, remained practically unchanged, but bands corresponding to vibrations of the ZnPcCl_x_ macrocycle appeared in the spectra of all hybrids.

**Figure 3:**
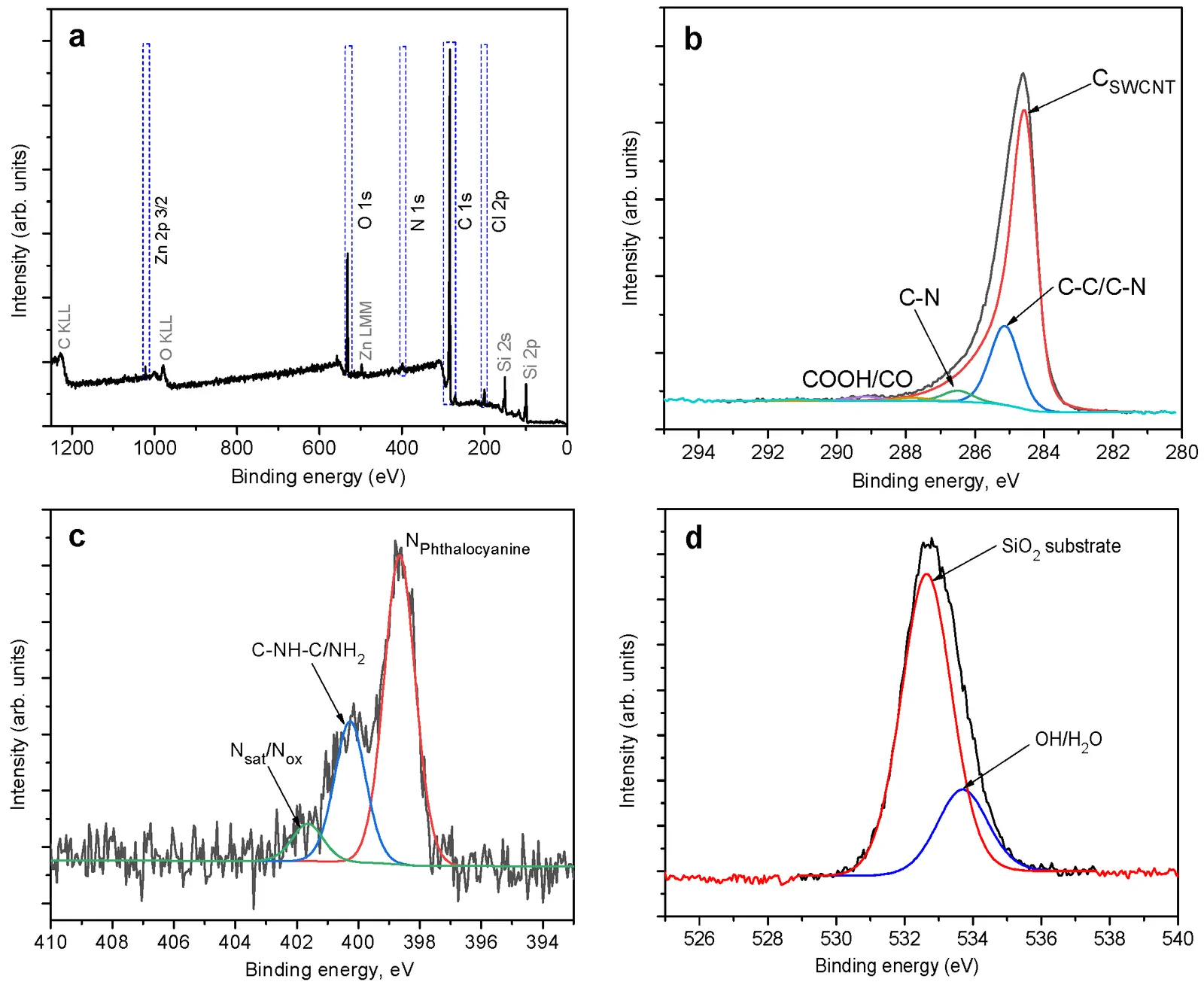
Survey (**a**), C1s (**b**), N1s (**c**), and O1s (**d**) XPS spectra of the SWCNT-NH_2_/ZnPcCl_8_ cov hybrid.

XPS analysis further verified the hybrid formation (Figure 3). XPS spectra were assigned by comparing the measured binding energies to literature values for metal phthalocyanines, halogenated phthalocyanines, aminated carbon nanomaterials, and their covalent conjugates with carbon nanotubes [34,35,36,37,38,39,40,41]. The survey spectrum of the hybrid (Figure 3a) shows C1s, N1s, O1s, Zn2p, and Cl2p peaks. The Cl2p (~200 eV) and Zn2p signals confirm the presence of chlorinated zinc phthalocyanine on the nanotube surface. The O1s peak is attributed to adsorbed species and minor oxygen-containing groups on the nanotubes. In the C1s spectrum of the SWCNT-NH_2_/ZnPcCl_8_ hybrid (Figure 3b), the dominant sp^2^-carbon peak is at 284.6 eV. Deconvolution reveals additional components at 285.2 eV (C–C/C–N), 286.5 eV (C–N), 287.8 eV (C=O), and 289.2 eV (O–C=O). The aromatic C–Cl carbon overlaps with the C–N region (286–287 eV), while the Cl2p peak at ~200 eV confirms covalent C–Cl bonds. Overall, the C1s profile supports the attachment of the chlorinated macrocycle to the nanotubes, with Cl2p and N1s peaks serving as the primary evidence for phthalocyanine incorporation.

The N1s spectrum of pristine SWCNT-NH_2_ shows a single peak at ~400.0 eV, corresponding to surface -NH_2_ groups (Figure S1). For the SWCNT-NH_2_/ZnPcCl_8_ hybrid, the N1s spectrum (Figure 3c) was deconvoluted into three components. The dominant peak at 398.6 eV, absent in pristine nanotubes, is assigned to the phthalocyanine macrocycle nitrogen. The peak at 400.4 eV corresponds to amine/secondary amine nitrogen, attributed to the C(macrocycle)-NH-C(nanotube) linker and residual unreacted amino groups. The component at 402 eV can be attributed to a shake-up satellite of the π → π* conjugated macrocycles and, possibly, to a small fraction of protonated or oxidized nitrogen observed in carbon and phthalocyanine systems in the range of 402–405 eV. The phthalocyanine nitrogen component (398.6 eV) dominates the N1s spectrum, while the amine/linker contribution (400.4 eV) is minor.

This aligns with the low degree of amine functionalization of the nanotubes and confirms that most of the observed nitrogen originates from the attached phthalocyanine. It is difficult to determine the exact cross-linking stoichiometry from the peak area ratios because the 400.4 eV component may include both linker and residual amine nitrogen, and overlap with a satellite signal. The shift of the amine nitrogen peak to 400.4 eV from the original 400.0 eV may indicate the conversion of primary amines to secondary arylamines attached to the electron-deficient chlorinated macrocycle. The peaks in the O1s XPS spectra of the SWCNT-NH_2_/ZnPcCl_8_ cov hybrid are mainly caused by the Si/SiO_2_ substrate and adsorbed water.

**Figure 4:**
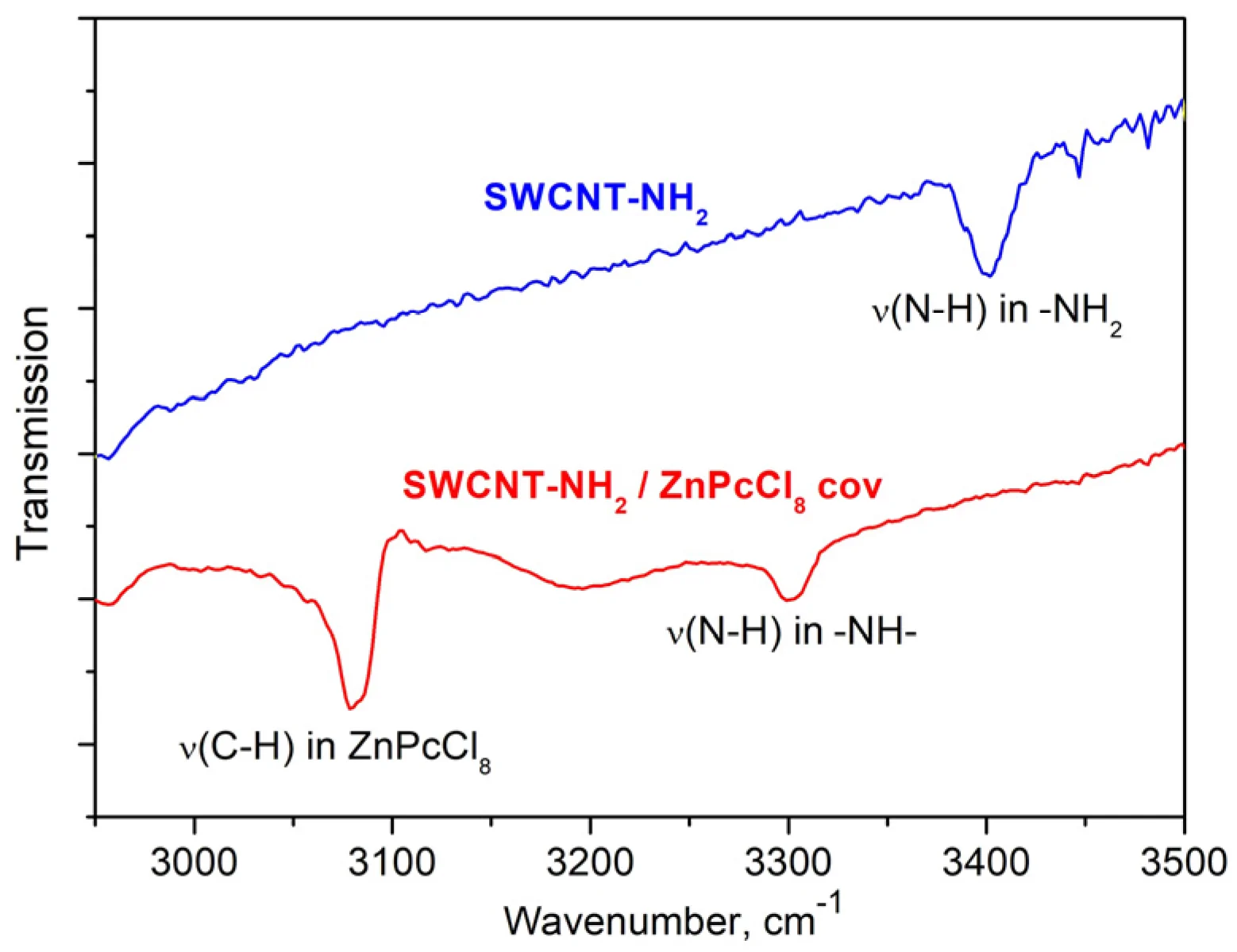
IR spectra of SWCNT-NH_2_ and SWCNT-NH_2_/ZnPcCl_8_ cov hybrid, measured in fluorinated oil (Fluorolube).

Due to the close proximity of the N1s XPS spectra speaks of the initial carbon nanotubes and the hybrid, it is difficult to thoroughly confirm the fact of covalent functionalization. Therefore, we recorded the IR spectra of the original nanotubes and the resulting hybrids. The IR spectra were recorded in fluorinated oil (Fluorolube^®^, Gabriel Performance Products, LLC, Harrison City, PA, USA) to avoid the interfering influence of water, which is always present in some amount in KBr. The spectra are presented in Figure 4. The spectrum shows that the SWCNT-NH_2_ spectra contain a band at 3400 cm^−1^, corresponding to the valence vibrations of the N-H bond in the NH_2_ group of primary amines. In the spectrum of the SWCNT-NH_2_/ZnPcCl_8_ cov hybrid, this band disappears completely, but a band is observed in the region of 3032 cm^−1^, which may indicate the formation of a C-NH-C bond.

**Figure 5:**
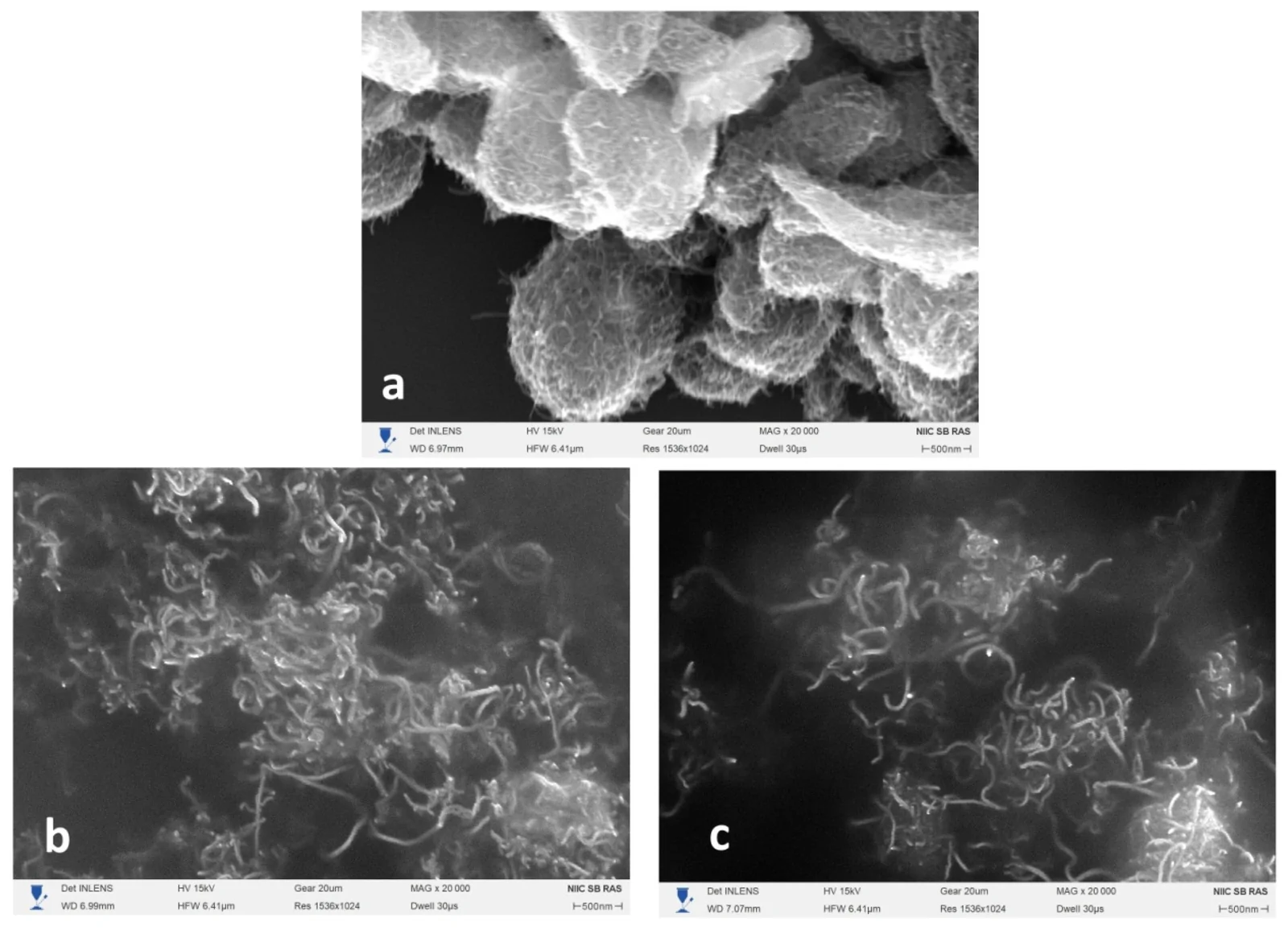
SEM images of pristine SWCNT-NH_2_ nanotubes (**a**), SWCNT-NH_2_/ZnPcCl_4_ cov (**b**), and SWCNT-NH_2_/ZnPcCl_8_ cov (**c**) hybrids.

Figure 5 displays representative SEM images of pristine SWCNT-NH_2_, SWCNT-NH_2_/ZnPcCl_4_ cov, and SWCNT-NH_2_/ZnPcCl_8_ cov hybrids. The images reveal that pristine SWCNT-NH_2_ strongly aggregates into dense clusters, consisting of compact bundles. In contrast, covalent functionalization with phthalocyanine molecules effectively mitigates this aggregation. Consequently, the hybrid materials exhibit a significantly more porous morphology, characterized by well-dispersed loosely packed bundles.

Table 1 presents the amount of phthalocyanine in the SWCNT-NH_2_/ZnPcCl_x_ cov hybrid materials, as determined using the ICP-AES method based on the metal content in the hybrid material. For comparative purposes, the phthalocyanine content in hybrids obtained via non-covalent functionalization of SWCNT with the same phthalocyanines is also included. ICP-AES quantification demonstrated that the covalent hybrids possess a significantly higher macrocycle loading compared to their non-covalent counterparts. A similar effect of increasing the degree of functionalization of nanotubes during their covalent modification with polyaromatic molecules through the click-chemistry reaction has also been reported in previous studies [6,42]. This increased degree of functionalization has several advantages, as it leads to a higher density of active sensing sites. This, in turn, enhances the sensitivity of the hybrid materials toward gaseous analytes.

**Figure 6:**
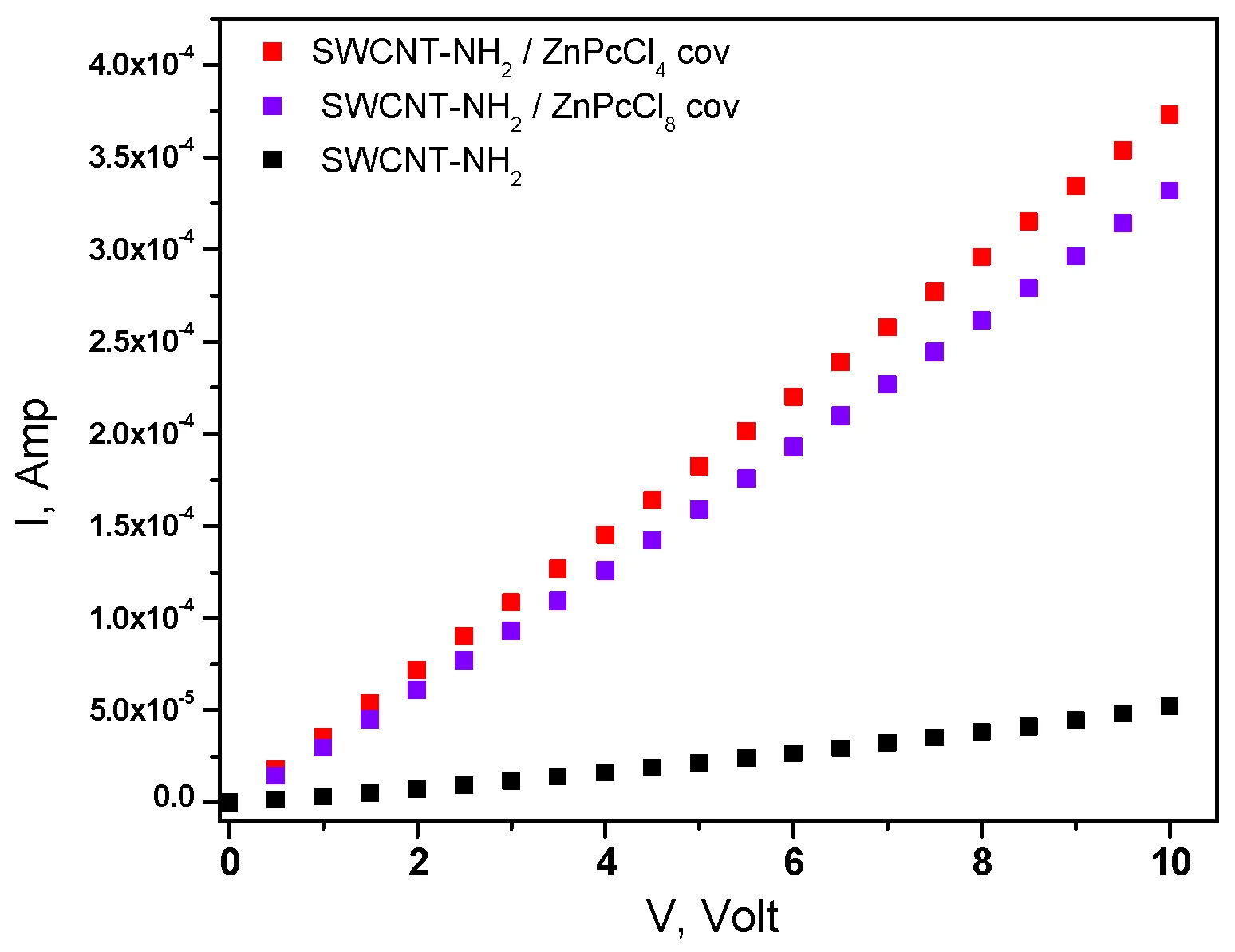
I–V dependences for layers of SWCNT-NH_2_/ZnPcCl_x_ cov hybrid materials.

Current–voltage (I–V) measurements (Figure 6) indicated that covalent functionalization enhanced the overall electrical conductivity of the hybrid films relative to bare nanotubes, likely due to improved inter-tube charge transport mediated by the conjugated macrocycles.

### 2.2. Chemiresistive Ammonia Sensing Performance

**Figure 7:**
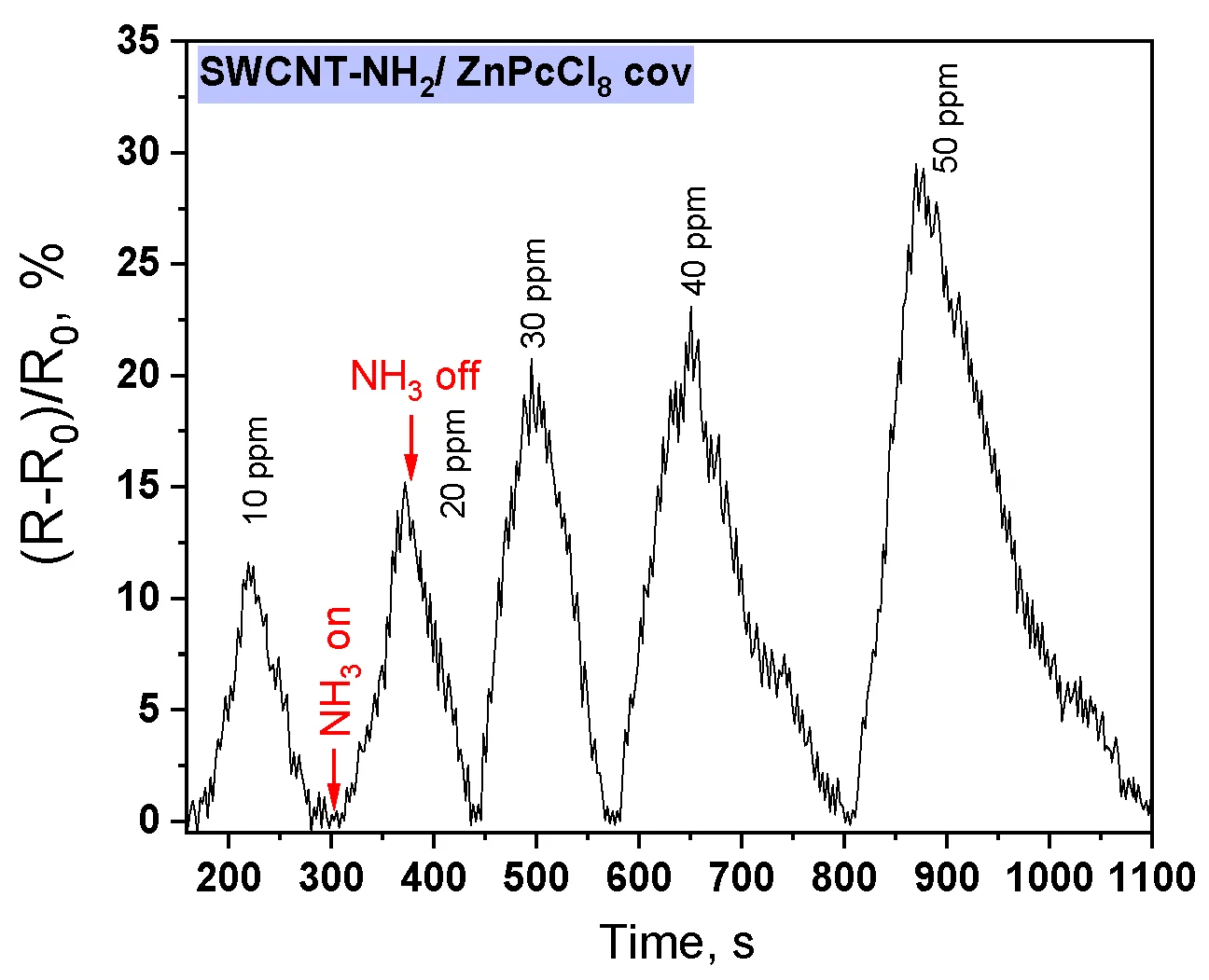
Sensor response of SWCNT-NH_2_/ZnPcCl_8_ cov layers to 10–50 ppm ammonia measured at room temperature and RH of 10 ± 5%.

Layers of all investigated hybrids exhibited fully reversible chemiresistive responses to NH_3_ (1–50 ppm) at room temperature. Upon exposure to ammonia, the electrical resistance of the layers increases, and when the air is purged, it returns to its initial baseline value. A typical curve of the response for SWCNT-NH_2_/ZnPcCl_8_ cov is shown in Figure 7 as a representative example. An increase in resistance in an ammonia atmosphere is characteristic of p-type semiconductors and is commonly observed in carbon nanotubes modified with various metal phthalocyanines [43].

**Figure 8:**
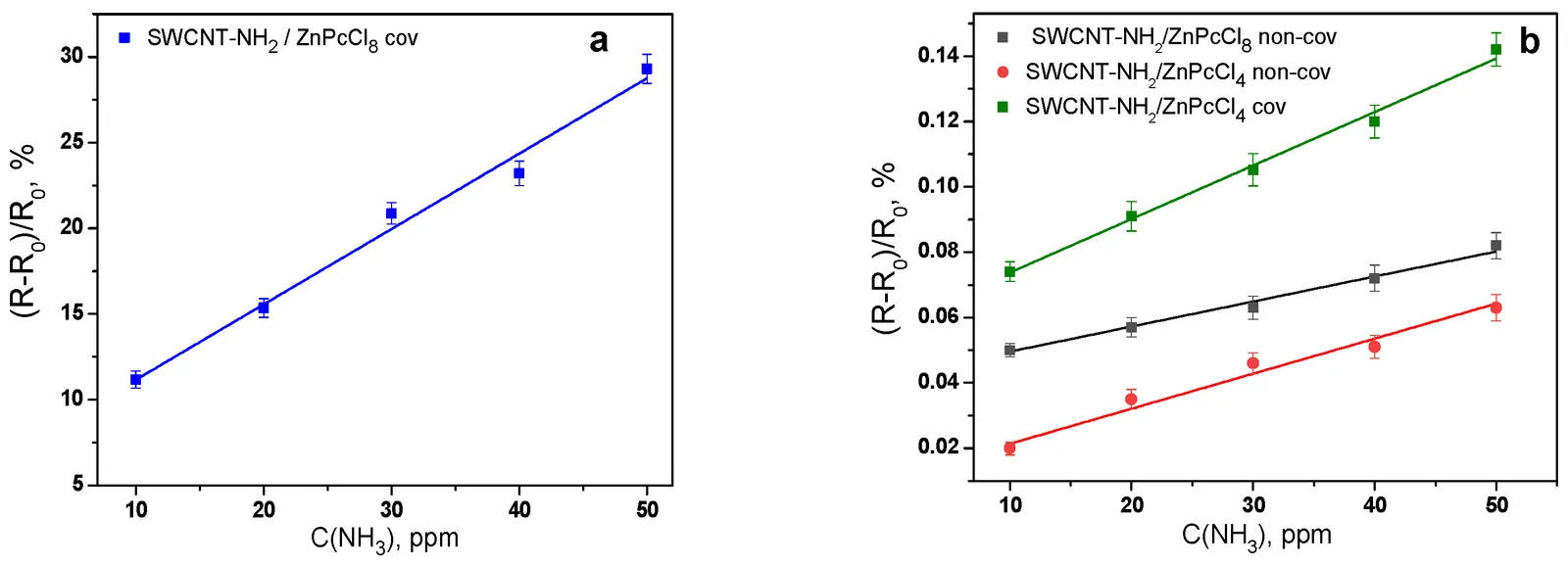
Dependence of the sensor response of the hybrid materials obtained through covalent and non-covalent functionalization of SWCNT-NH_2_ with ZnPcCl_x_ on the concentration of ammonia (10–50 ppm): SWCNT-NH_2_/ZnPcCl_8_ cov (**a**), SWCNT-NH_2_/ZnPcCl_4_ cov, SWCNT-NH_2_/ZnPcCl_8_ non-cov (**a**) and SWCNT-NH_2_/ZnPcCl_4_ non-cov (**b**).

SWCNT-NH_2_ functionalized with phthalocyanines consistently delivered higher responses than pristine SWCNT-NH_2_. The dependencies of the sensor response of hybrid materials, obtained via both covalent and non-covalent functionalization of SWCNT-NH_2_, on the concentration of ammonia (10–50 ppm) are presented in Figure 8a and Figure 8b, respectively.

To assess the sensor performance, the limits of detection (LODs), response times, and recovery times were calculated for all materials under investigation and summarized in Table 2. All of the layers have relatively short response and recovery times. Covalent functionalization improves LODs. The LOD of the SWCNT-NH_2_/ZnPcCl_4_ cov hybrid reaches 0.8 ppm, while in the SWCNT-NH_2_/ZnPcCl_8_ cov layers, the LOD is 0.7 ppm. The pristine SWCNT-NH_2_ sensitivity to NH_3_ is extremely low. Non-covalent functionalization provides only a minor gain (0.06% for ZnPcCl_4_ and 0.08% for ZnPcCl_8_), while materials obtained via covalent functionalization show an increase in sensitivity, especially for SWCNT-NH_2_/ZnPcCl_8_ (Table 2). The higher sensitivity of covalent hybrids compared to non-covalent ones correlates with a high content of phthalocyanines in them, according to ICP-AES data (Table 1).

Among the covalently grafted series, SWCNT-NH_2_/ZnPcCl_8_ cov demonstrated the strongest signal, which directly correlated with its higher phthalocyanine content (Table 1). A clear structure–property trend emerged: octa-halogenated derivatives (ZnPcCl_8_) outperformed the tetra-halogenated analogue. The best sensing layer, SWCNT-NH_2_/ZnPcCl_8_ cov, achieved an LOD of 0.7 ppm and low response/recovery times (60/65 s at 10 ppm NH_3_).

### 2.3. Environmental Stability and Cross-Selectivity

**Figure 9:**
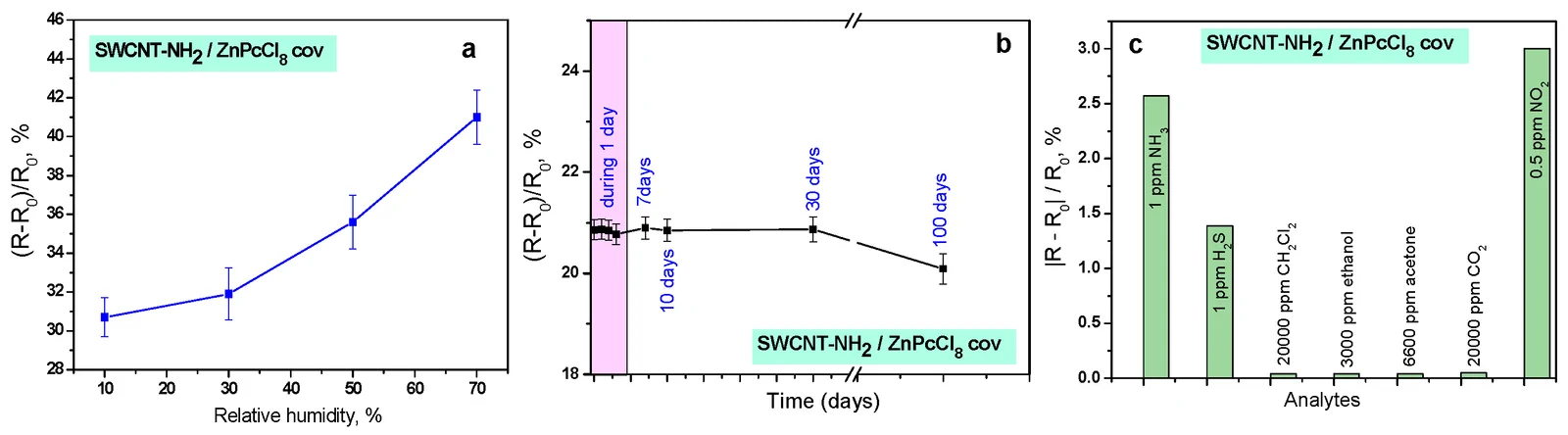
(**a**) Sensor response of SWCNT-NH_2_/ZnPcCl_8_ cov to 50 ppm NH_3_ measured at different RH. (**b**) Repeatability and stability of SWCNT-NH_2_/ZnPcCl_8_ cov layers. (**c**) Sensor response of SWCNT-NH_2_/ZnPcCl_8_ cov to NH_3_ (1 ppm), H_2_S (1 ppm), CO_2_ (20,000 ppm), NO_2_ (0.5 ppm), dichloromethane (20,000 ppm), ethanol (3000 ppm), and acetone (6600 ppm).

The influence of operating temperature and relative humidity (RH) on sensor performance was systematically evaluated. Increasing the operating temperature up to 60 °C had negligible impact on the baseline or response value. Elevating RH to 70% increased the NH_3_ response of SWCNT-NH_2_/ZnPcCl_8_ cov by approximately 1.3 times (Figure 9a), likely due to competitive water–analyte adsorption dynamics. Below 40% RH, sensor characteristics remained highly stable.

The hybrid layers displayed excellent cycle-to-cycle reproducibility and stability for no less than one month (Figure 9b) of storage under ambient conditions. Furthermore, the sensor exhibited strong discrimination against CO_2_ and common volatile organic compounds (VOCs) (Figure 9c).

However, noticeable cross-sensitivity toward H_2_S and NO_2_ was observed, indicating these gases as primary interferents for ammonia quantification in complex atmospheres (Figure 9c). Because these gases are highly reactive and possess strong redox or acid-base characteristics, they can interact with the central metals and π-systems of the phthalocyanines as well as with the carbon nanotube framework, indicating them as primary interferents for ammonia quantification in complex, multi-gas atmospheres. While localized acid-base or redox interactions between the Zn center and gases like H_2_S (a soft Lewis base) or NO_2_ can occur at the molecular level, our quantitative ICP-AES data (Table 1) indicates that the phthalocyanine loading is quite low. Because the Zn centers are sparse and isolated, their overall contribution to the conductive pathway and transduction process is likely to be statistically insignificant compared to that of the highly accessible SWCNT network. The strong charge-transfer interactions between the interfering gases (H_2_S and NO_2_) and the carbon nanotube framework may play a more significant role. NO_2_, a strong oxidizing agent that extracts electrons from the p-type SWCNT network, increases the conductivity of the hybrid material. H_2_S can act as a reducing agent, decreasing the conductivity. To demonstrate the interfering effect of H_2_S, the sensor response was measured in the mixture of NH_3_ and H_2_S in air (Figure S2). It has been shown that adding H_2_S to the tested gas mixture not only increases the sensor response but also affects the response time and recovery time of the sensor. A more detailed study of the possibility of detecting ammonia in complex gas mixtures is beyond the scope of this work and is the subject of separate investigation.

Some important sensing characteristics of SWCNT-NH_2_/ZnPcCl_8_ cov layers toward NH_3_ described in this work are compared with the other sensors based on carbon nanomaterials functionalized with metal phthalocyanines, which have been described in the literature (Table 3). We find that the sensing layers based on SWCNT-NH_2_/ZnPcCl_8_ are quite competitive with the active layers based on hybrid materials that have been reported in the literature.

### 2.4. Quantum–Chemical Modeling

**Figure 10:**
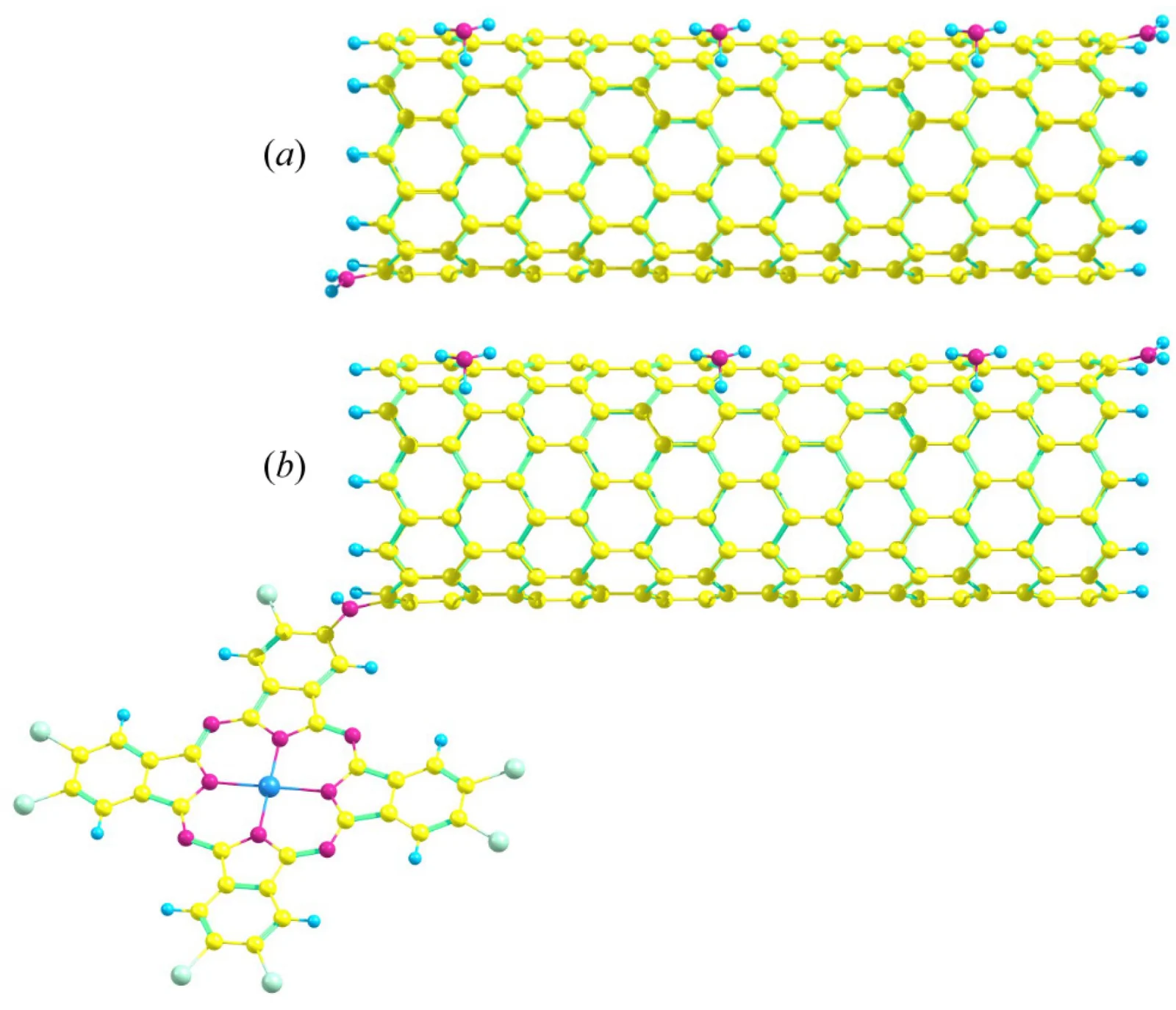
Optimized geometric structures of SWCNT (**a**) and SWCNT-NH_2_/ZnPcCl_8_ (**b**) with three NH_3_ molecules adsorbed on the carbon surface. carbon atoms are colored yellow, nitrogen atoms red, and hydrogen atoms blue.

To interpret the experimental data on the sensor response of the SWCNT-NH_2_/ZnPcCl_x_ hybrids to ammonia, quantum–chemical calculations of the geometric and electronic structure of the hybrids and their species with NH_3_ molecules were performed. The semiconductor nanotube was modeled as a SWCNT(10,0) cluster consisting of six unit cells, with the dangling bonds at the cluster edges terminated by hydrogen atoms and two NH_2_ groups (Figure 10a). The hybrid structures were simulated by the covalent attachment of ZnPcCl_4_ and ZnPcCl_8_ macrocycles to one of the surface amino groups via an NH-bridge (hereafter SWCNT-NH_2_/ZnPcCl_4_ and SWCNT-NH_2_/ZnPcCl_8_), as described above. The interaction with ammonia was investigated using models containing three NH_3_ molecules adsorbed directly on the carbon surface of the nanotube (Figure 10b).

The computational model represents a simplification, as the phthalocyanine macrocycle is attached to the edge of the finite SWCNT cluster rather than to the pristine sidewall. However, this is physically justified by experimental data. XPS analysis revealed a low initial amino-functionalization degree (0.1 at.% N), indicating that macrocycles attach at isolated defect sites, and IR spectroscopy confirmed complete conversion of primary amines into C-NH-C linkers (Figure 4), so macrocycles are spatially isolated on the sp^2^-framework. The edge location is representative of commercial amino-functionalized SWCNTs, where functionalization occurs predominantly at defect sites at tube ends and curvature points [52,53,54]. The covalent NH-bridge creates sp^3^-hybridization, acting as an electronic buffer decoupling the macrocycle from edge effects. The p-doping effect from electron-withdrawing chlorine substituents is transmitted through the π-system and NH-bridge to the sp^2^-framework, fundamentally independent of attachment location. Finite SWCNT clusters with hydrogen-terminated edges correctly reproduce electronic properties of infinite nanotubes [55,56,57]. Thus, the localized cluster approach is physically adequate for elucidating how peripheral halogenation governs charge transfer from NH_3_, and the edge location does not affect qualitative conclusions.

The calculations showed that the covalent functionalization of the nanotube via the NH-bridge practically does not affect the NH_3_ adsorption energy. The energy remains in a narrow range between −0.126 and −0.124 eV (Table 4). However, the attachment of the phthalocyanine molecules reduces the total negative charge of the carbon framework (*q*_0_), which is equivalent to an increase in hole concentration. This finding is in good agreement with the experimental data as the adsorption of ammonia (an electron donor) leads to a decrease in hole concentration and an increase in the resistance of *p*-type nanotubes. Analysis of the calculated effective charges reveals that an increase in the number of peripheral halogen atoms in the phthalocyanine leads to a further depletion of electrons in the carbon framework (an increase in the hole concentration), as evidenced by the decrease in the absolute value of *q*_0_ when going from SWCNT-NH_2_/ZnPcCl_4_ to SWCNT-NH_2_/ZnPcCl_8_ (Table 4). This effect can be attributed to the high electronegativity of the halogen atoms, which act as strong electron-withdrawing groups, pulling electron density from the *π*-system of the phthalocyanine macrocycle. This electron depletion is subsequently transferred to the carbon framework through the covalent NH-bridge, effectively acting as a *p*-dopant for the nanotube.

The adsorption of ammonia is accompanied by the transfer of negative charge to the carbon framework. This causes a decrease in the concentration of the majority charge carriers, which in turn leads to an increase in electrical resistance. In hybrids, this effect is amplified and correlates with the degree of halogenation of the macrocycle: the relative decrease in the hole concentration in the series SWCNT → SWCNT-NH_2_/ZnPcCl_4_ → SWCNT-NH_2_/ZnPcCl_8_ is 0.96%, 1.44%, and 1.57%, respectively (Table 4). Notably, the absolute charge transfer from NH_3_ molecules to the carbon framework remains nearly constant across the series (|Δ*q*| ≈ 0.023–0.024 *e* for all hybrids, Table 4). Therefore, the enhanced relative decrease in hole concentration (Δ*p*/*p*_0_) is not due to stronger analyte binding but rather to the lower baseline hole concentration |*q*_0_| in heavily halogenated systems. In such electron-depleted frameworks, even a small additional charge transfer from NH_3_ induces a proportionally larger relative change in the majority carrier concentration, thereby amplifying the resistive signal, and this theoretical trend perfectly matches the experimentally observed increase in the sensor response when transitioning from tetra-substituted (ZnPcCl_4_) to the octa-substituted (ZnPcCl_8_) phthalocyanines.

## 3. Materials and Methods

### 3.1. Preparation of Hybrid Materials

Amino-functionalized single-walled carbon nanotubes (SWCNT-NH_2_, SWCNT Type 6, Length 5–30 µm, OD 1–2 nm, 99%) were purchased from Sisco Research Laboratories Pvt. Ltd. (SRL, Maharashtra, India). According to the XPS analysis, they contain 0.1 at.% nitrogen on their surface.

ZnPcCl_x_ derivatives were synthesized according to previously published techniques [58]. Hybrid materials were obtained through two different methods. One method involved non-covalent functionalization of SWCNT-NH_2_ with ZnPcCl_x_. Non-covalent hybrids (SWCNT-NH_2_/ZnPcCl_x_ noncov) were created by dispersing SWCNT-NH_2_ in tetrahydrofuran (THF, Reachim, Russia) and then adding the target ZnPcCl_x_ in small drops while stirring continuously. The mixture was sonicated for 60 min, then centrifuged repeatedly and washed with fresh THF to remove any unbound macrocycles.

Covalent hybrids (SWCNT-NH_2_/ZnPcCl_x_ cov) were synthesized through the nucleophilic substitution of peripheral halogen atoms in the phthalocyanine ring by surface -NH_2_ groups, with concomitant release of HCl, according to the previously described technique [32]. In a typical procedure, SWCNT-NH_2_ (5.0 mg) and ZnPcCl_x_ (3.5 mg) were suspended in anhydrous dimethylformamide (DMF (SRL, Maharashtra, India), 3 mL) by means of sonication. Triethylamine (Maclin, Shanghai, China, 100 μL) was added as a base, and the mixture was degassed using three freeze–pump–thaw cycles. The reaction flask was sealed and heated in an oil bath at 100 °C for 24 h under stirring. After cooling, the black product was isolated via centrifugation (3500 rpm, 5 min, repeated three times), thoroughly washed subsequently with acetone and ethanol (Reachim, Russia), then dried at >50 °C for 12 h.

### 3.2. Characterization of Hybrid Materials

Raman spectra were recorded via a LabRAM HR Evolution spectrometer (Horiba Ltd., Kyoto, Japan) with a CCD Symphony (Jobin Yvon, Longjumeau, France) detector with 2048 horizontal pixels. The 514 nm line of an Ar-laser (CVI Melles Griot, Carlsbad, CA, USA) was used for the spectral excitation. The laser power was typically less than 0.1 mW. The spectra were measured in backscattering collection geometry with a Raman microscope (Horiba Ltd., Kyoto, Japan).

IR spectra of the samples were recorded with a Vertex 80 FTIR spectrometer (Vertex, Ettlingen, Germany) in fluorinated oil (Fluorolube^®^).

Zinc content in the hybrids was determined via inductively coupled plasma atomic emission spectrometry ICP-AES (iCAP-6500 Duo, ThermoFisher Scientific, Singapore). Samples were dissolved in concentrated H_2_SO_4_ and diluted to 10 mL with deionized water. Analysis parameters: 1150 W power; Ar flow rates: 0.70 (nebulizer), 0.50 (auxiliary), and 12 L/min (cooling).

Scanning electron microscopy (SEM) of hybrid materials was carried out on a CIQTEK SEM 5000 (CIQTEK Ltd., Hefei, Anhui, China) microscope at an accelerating voltage of 15 kV.

Hybrid materials were investigated using X-ray photoelectron spectroscopy (XPS) on a FlexPS spectrometer (SPECS Surface Nano Analysis GmbH, Berlin, Germany). The spectrometer was equipped with a Phoibos 150 analyzer (SPECS Surface Analysis and Computer Technology, Berlin, Germany) using a monochromatic Al Kα source, with an excitation energy of 1486.71 eV. XPS survey spectra were recorded at a pass energy of 50 eV with a 0.5 eV step size (1 scan). High-resolution spectra were acquired using a pass energy of 20 eV and a 0.05 eV step size, with 5 scans for C1s and 20 scans for N1s, Cl2p, and Zn2p spectra. The XPS core-level spectra were analyzed using the Casa XPS software, Version 2.3.15 (Casa Software Ltd., Teignmouth, UK). The spectra were fitted using a Gaussian/Lorentzian product function and a Shirley-type background. Commercial Si(100) substrates covered with native oxide were used as substrates for both SEM and XPS studies. The substrates were pre-cleaned with acetone in an ultrasonic bath, rinsed with deionized water, and then dried with pure air. The samples of carbon nanotubes and hybrids were deposited via drop-casting of their suspensions in dichloromethane. Charge correction of the XPS spectra was performed by setting the principal C 1s core level peak, attributed to sp^2^-hybridized carbon of nanotubes, to 284.6 eV.

### 3.3. Study of Chemiresistive Sensor Response

For gas sensing evaluation, the hybrid dispersions were deposited onto glass substrates patterned with platinum interdigitated electrodes (IDEs) via drop-casting techniques (30 μL suspension in dichloromethane). The electrical resistance of the phthalocyanine films was measured using a Keithley 236 electrometer (Keithley Instruments, Inc., Solon, OH, USA), with a constant direct current voltage of 10 V applied. All gas sensing experiments were conducted at room temperature. The change in resistance during gas addition was measured using a Keithley electrometer. The relative sensor response was calculated as (R_i_ − R_0_)/R_0_ × 100, where R_0_ and R_i_ denote the baseline resistance in air and the steady-state resistance upon exposure to a specific analyte concentration, respectively.

The gas sensing tests were performed in a custom-made measurement chamber with an internal volume of 70 mL. The source of NH_3_ was a commercial gas cylinder containing 1% NH_3_ diluted in dry argon (“Chistye Gasy+”, Novosibirsk, Russia). Dry air was used as the diluting and purging gas. To achieve the required NH_3_ concentrations (10–50 ppm), the 1% NH_3_/Ar gas and dry air were precisely mixed using electronic mass flow controllers. The gases were mixed in the gas lines prior to entering the measurement chamber to ensure a homogeneous analyte–air mixture. The total gas flow rate was maintained at a constant 300 mL/min during all dynamic measurements. Prior to testing, the sensor was exposed to pure air for 5–10 min until the baseline resistance stabilized. Most gas sensing experiments were conducted in a dynamic flow mode. The NH_3_/air mixture was introduced into the chamber for a fixed exposure time of 30 s, followed by purging with pure air until the initial resistance was recovered.

To determine the response and recovery times of the sensors, a static mode was employed. In this regime, the airflow was passed through the chamber until the film resistance reached a constant baseline. Then, the chamber valves were closed, and a specific concentration of NH_3_ was injected directly into the cell. The resistance was continuously recorded until a saturation value was reached to calculate the response time. Afterward, the chamber was opened and purged with a continuous air stream to record the recovery time.

To evaluate the effect of moisture on sensor performance, a wet carrier gas was prepared by bubbling the dry air through a flask containing distilled water at room temperature. The relative humidity (RH) inside the measurement cell was continuously monitored and controlled using a commercially available humidity meter (MPE-202.013, Merapribor, Russia).

### 3.4. Computational Details

Quantum–chemical calculations were performed using the self-consistent charge density-functional tight-binding (SCC-DFTB) method as implemented in the DFTB+ software package (version 24.1) [59,60], with the 3OB parameter set [61,62] and DFT-D3 dispersion correction [63,64]. Geometry optimization was carried out until the forces acting on the atoms did not exceed 0.0001 a.u. The average adsorption energy (*E_ads_*) of each gas molecule was estimated as one-third of the difference between the total energies of the entire aggregate, the SWCNT(10,0) cluster (or the corresponding hybrid), and three isolated NH_3_ molecules. The relative change in the charge carrier concentration in the carbon nanotube upon ammonia adsorption was determined as ∆*p*/*p*_0_ = |*q*_0_ − *q*|/|*q*_0_|, where *q*_0_ and *q* are the sums of the Mulliken effective charges [65,66] of the carbon atoms before and after the adsorption of NH_3_ molecules, respectively.

## 4. Conclusions

In this work, new hybrid materials containing single-walled carbon nanotubes (SWCNT-NH_2_) covalently functionalized with chloro-substituted zinc phthalocyanines (ZnPcCl_4_ and ZnPcCl_8_) were synthesized and evaluated, which were used to determine the ammonia content at room temperature. Covalent attachment of macrocycles via nucleophilic substitution turned out to be very advantageous compared to non-covalent functionalization, which led to a significantly higher degree of functionalization of nanotubes, improved morphological dispersion, and increased electrical conductivity. A significant improvement in sensor response was observed as the degree of halogenation increased. The response of the octa-substituted ZnPcCl_8_ hybrid was more than 10 times higher than that of the tetra-substituted ZnPcCl_4_ analogue and over 100 times higher than those of non-covalent functionalized hybrids. The SWCNT-NH_2_/ZnPcCl_8_ covalent hybrid proved to be the optimal sensing material, with a low limit of detection of 0.7 ppm and fast response and recovery times of 60 and 65 s at 10 ppm, respectively. The optimized sensor demonstrated excellent cycle-to-cycle reproducibility and long-term stability, retaining its performance for more than a month. It showed strong discrimination against CO_2_ and common volatile organic compounds. However, cross-sensitivity to hydrogen sulfide and nitrogen dioxide was identified as a potential source of interference in complex gas mixtures.

Quantum–chemical modeling elucidated the underlying sensing mechanism. Quantum–chemical calculations revealed that functionalizing SWCNTs with halogenated ZnPc macrocycles does not alter NH_3_ adsorption energy but induces a strong p-doping effect, increasing the baseline hole concentration due to the electron-withdrawing nature of the halogens. Heavily halogenated hybrids exhibit an enhanced relative decrease in hole concentration upon NH_3_ exposure because their highly electron-depleted baseline magnifies the impact of the constant analyte charge transfer.

This work not only provides a high-performance sensor material but also establishes a clear theoretical framework for designing future carbon nanomaterial/phthalocyanine hybrids by tuning the electron-withdrawing capabilities of peripheral substituents.

## Figures and Tables

**Table 1:** ZnPcCl_x_ content in SWCNT-NH_2_/MPcCl_x_ hybrid materials (mmol/g) (the error of a single determination does not exceed 10%).

Hybrid Material	ν_MPcClx_/m_hybr_
SWCNT-NH_2_/ZnPcCl_8_ cov	0.199
SWCNT-NH_2_/ZnPcCl_4_ cov	0.089
SWCNT-NH_2_/ZnPcCl_8_ noncov	0.011
SWCNT-NH_2_/ZnPcCl_4_ noncov	0.007

**Table 2:** Sensitivity, limits of detection (LODs), response times, and recovery times for all materials under investigation.

Material	LOD, ppm	Response/Recovery Time, s (10 ppm)	Sensitivity, % (50 ppm)
SWCNT-NH_2_	1	55/180	0.01
SWCNT-NH_2_/ZnPcCl_4_ noncov	1	65/100	0.06
SWCNT-NH_2_/ZnPcCl_8_ noncov	0.9	50/120	0.08
SWCNT-NH_2_/ZnPcCl_4_ cov	0.8	35/130	0.15
SWCNT-NH_2_/ZnPcCl_8_ cov	0.7	60/65	30

**Table 3:** Characteristics of sensors based on carbon nanomaterials functionalized with metal phthalocyanine derivatives.

Hybrid	Concentration Range, ppm	Response/Recovery Time, s	Sensitivity, %	Ref.
CuTSPc@3D-(N)GF/PEDOT-PSS	1–1000	138/63 (200 ppm)	8 (50 ppm)	[44]
TFPCoPc/MWCNT	0.1–200	46/450 (50 ppm)	26 (50 ppm)	[45]
CuPc-3/MWCNTs	0.6–80	360/175 (2.5 ppm)	40.5 (80 ppm)	[46]
PbPc/SWCNT-COOH	0.3–80	n/a	~30 (80 ppm)	[47]
ZnPc/MWCNT	0–52	180/~1800 (52 ppm)	n/a	[48]
PPy-TcCoPc/MWCNT	0.05–500	11/47 (5 ppm)	13.2 (5 ppm)	[49]
SWCNT/CoPc cov	0.1–50	30/20 (1 ppm)	0.8 (50 ppm)	[50]
SiPc/SWCNT cov	1–50	Na/100 (5 ppm)	~1 (50 ppm)	[51]
SWCNT-NH_2_/ZnPcCl_8_ cov layers	1–50	60/65 (10 ppm)	30 (50 ppm)	This work

CuTSPc—copper phthalocyanine-3,4′,4″,4‴-tetrasulfonic acid tetrasodium salt, PEDOT-PSS—poly(3,4-ethylenedioxythiophene)-poly(styrenesulfonate), 3D-(N)GF—three-dimensional nitrogen-doped graphene, TFPCoPc—2,9,16,23-tetrakis(2,2,3,3-tetrafluoropropoxy) metal(II) phthalocyanine, CuPc-3—copper,tetra-α-isopentyloxyphthalocyanine, PbPc—lead 2,9,16,23-tetra-iso-pentyloxyphthalocyanine, PPy-TcCoPc—tetra-β-carboxylate cobalt phthalocyanine tetrasodium salt bridged polypyrrole, CoPc—cobalt phthalocyanines containing 16 terminal ethynyl groups, SiPc—axially-bis(propynoxy)phthalocyaninato silicon(IV), n/a - not available.

**Table 4:** Average ammonia adsorption energies (*E_ads_*), total Mulliken effective charges of the carbon atoms in the clusters before (*q*_0_) and after (*q*) the adsorption of three NH_3_ molecules, and the corresponding relative decrease in hole concentration (Δ*p*/*p*_0_).

Cluster	*E_ads_*, eV	*q*_0_, *e*	*q*, *e*	Δ*p*/*p*_0_, %
SWCNT	−0.126	−1.6162	−1.6317	0.96
SWCNT-NH_2_/ZnPcCl_4_	−0.126	−1.6043	−1.6275	1.44
SWCNT-NH_2_/ZnPcCl_8_	−0.124	−1.5520	−1.5764	1.57

## Data Availability

The original contributions presented in this study are included in the article/Supplementary Materials. Further inquiries can be directed to the corresponding author.

## Supplementary Materials

The following supporting information can be downloaded at: https://www.mdpi.com/article/10.3390/ijms27188275/s1.
