# Supplementary material for: Modification of Single-Walled Carbon Nanotubes with Halogen-Substituted Metal Phthalocyanines for Chemiresistive Ammonia Detection
Source: Int J Mol Sci. 2026 Sep 17;27(18):8275. doi: 10.3390/ijms27188275 (PMC13607276; doi:10.3390/ijms27188275)
Supplement: Supplementary file 1 [file ijms-27-08275-s001.zip › ijms-4480261-supplementary.pdf]

Supporting Information

Modification of Single-Walled Carbon Nanotubes with Halogen-Substituted Metal Phthalocyanines for Chemiresistive Ammonia Detection

Darya Klyamer[1], Pavel Krasnov[2], Victoria Volchek[1], Evgeny Maksimovskiy[1], Anastasiya Fedorenko[1], Victoria Ivanova[1] and Tamara Basova[1]

[1]Nikolaev Institute of Inorganic Chemistry, Siberian Branch of Russian Academy of Sciences, 3, Acad. Lavrentiev Ave., Novosibirsk, 630090, Russian Federation

[2]International Research Center of Spectroscopy and Quantum Chemistry, Siberian Federal University, 26 Kirensky St., Krasnoyarsk, 660074, Russian Federation

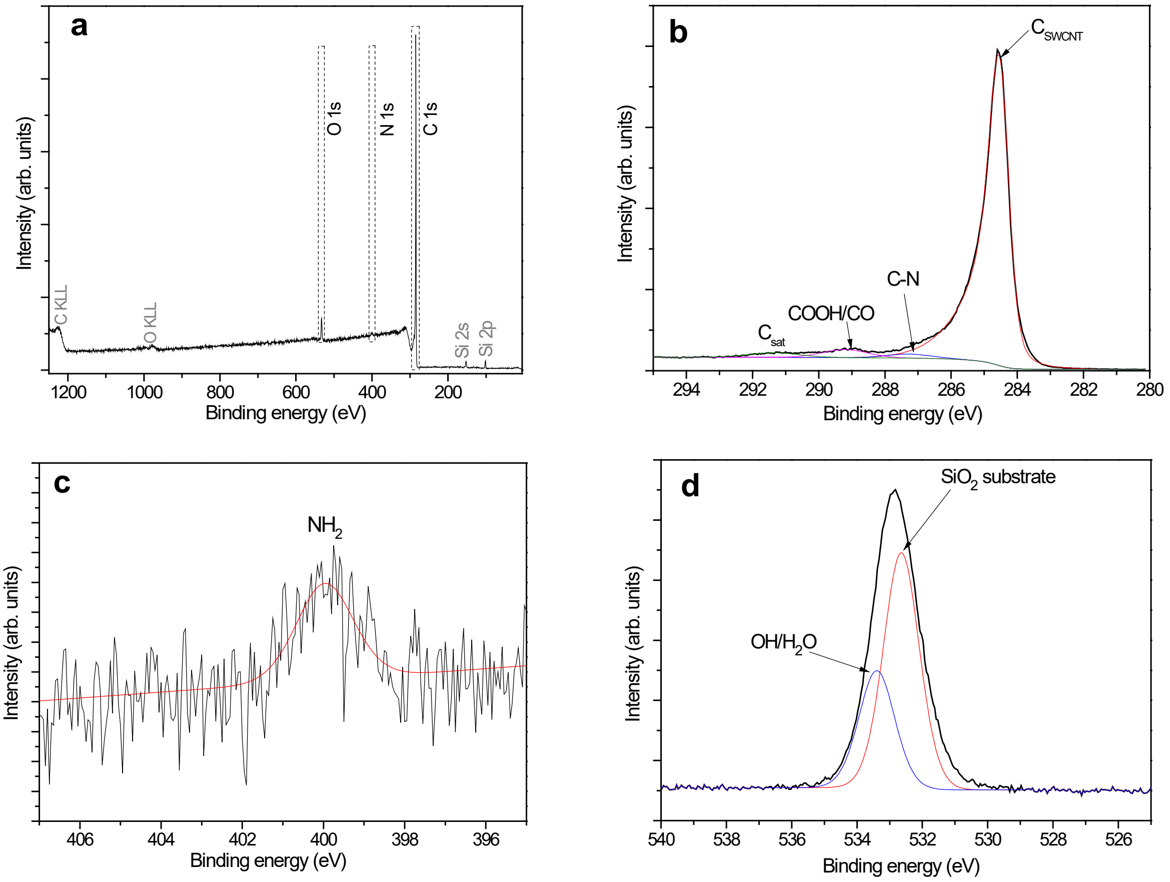

Figure S1. Survey (a), C1s (b), N1s (c), and O1S (d) XPS spectra of SWCNT-NH_2.

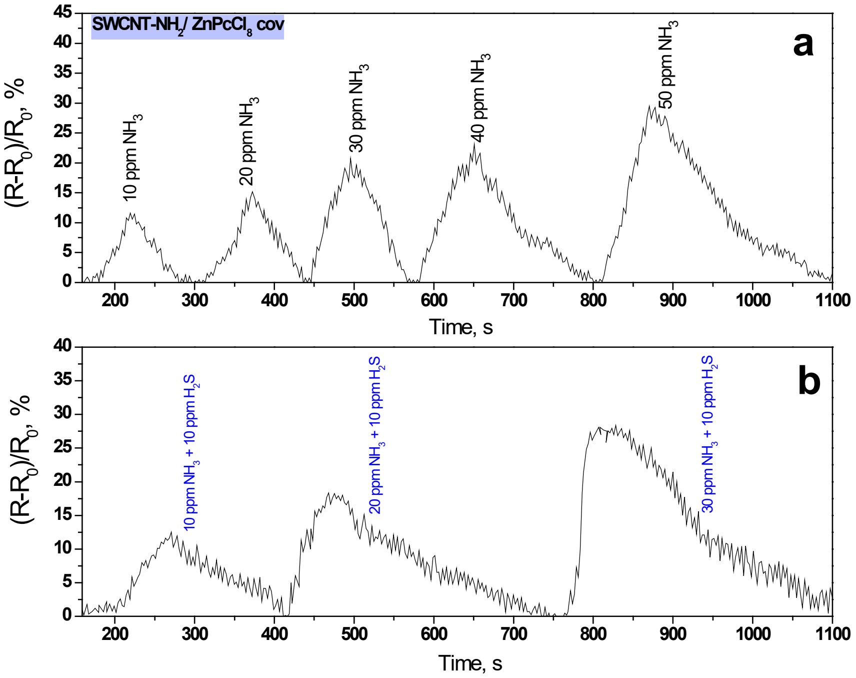

Figure S2. Sensor response of SWCNT-NH_2/$ZnPcCl_8$ cov layers to 10-50 ppm NH_3 (a) and to the mixture of NH_3 (10-30 ppm) and H_2S (10 ppm) in air.
